# Role of Omega-3 Fatty Acid Infusion in Surgical Outcomes of Perforation Peritonitis Patients: A Randomized Controlled Trial

**DOI:** 10.7759/cureus.23950

**Published:** 2022-04-08

**Authors:** Sadhasivam Ramasamy, Sudhir Jain, Ronal Kori, Shivani Atri, Chandra B Singh

**Affiliations:** 1 General Surgery, Milton Keynes University Hospital, Milton Keynes, GBR; 2 General Surgery, Employee's State Insurance Corporation (ESIC) Medical College and Hospital, Faridabad, IND; 3 General Surgery, Kettering General Hospital, Kettering, GBR; 4 General Surgery, Maulana Azad Medical College, Delhi, IND

**Keywords:** nutrition, post operative outcome, immune-enhancing, perforation peritonitis, omega-3 fatty acids

## Abstract

Background

Perforation peritonitis is associated with a high rate of morbidity and mortality in spite of advances in antibiotics and surgical techniques. The Omega-3 fatty acid is an immune-enhancing essential fatty acid that has been found to have anti-inflammatory properties, which help in quicker recovery. The present study examined the role of Omega-3 fatty acid infusion in the surgical outcome of perforation peritonitis.

Methods

Three hundred consecutive patients in the age group of 18-70 years operated for perforation peritonitis were included in this study. Patients in the study group received Omega-3 fatty acid emulsion postoperatively while those in the control group received a placebo. The groups were compared with respect to clinical and biochemical parameters.

Results

The Omega-3 fatty acid helped in reducing postoperative complications. The incidence of postoperative pyrexia (22.67% versus 82.67%), chest infection (6% versus 31.33%), and complete wound dehiscence (12% versus 34%) was significantly less in the study group compared to the control group.

There was a 4.5-day difference in overall length of stay, favoring the study group who were on Omega-3 fatty acids (LOS 8.06 vs. 12.65 days). There was no mortality in the study group compared with 17 deaths (11.3%) in the control group.

Conclusion

Postoperative perforation peritonitis patients receiving Omega-3 fatty acids are at a lower risk of developing postoperative complications, have a shorter duration of hospital stay, and have lower morbidity and mortality.

## Introduction

Perforation peritonitis is a common surgical emergency that bears high morbidity and mortality, despite the use of advanced surgical techniques and aggressive antibiotic therapy [1]. The majority of these cases are treated by laparotomy. The complications associated with laparotomy include surgical complications, such as wound dehiscence and postoperative collection/anastomotic leak, as well as other complications such as postoperative chest infection, myocardial infarction, deep vein thrombosis, and pulmonary embolism. If the duration of surgery is longer, there is a high risk of postoperative ileus. Recovery after a laparotomy depends on the etiology, duration of surgery, nutritional status, the American Society of Anesthesiologists (ASA) physical status classification system, and co-morbidities such as anemia and chronic obstructive pulmonary disease (COPD). The morbidity and mortality can be minimized by appropriate perioperative care, which includes multimodal analgesia, fluid and electrolyte management, correction of surgical stress, early mobilization, and optimization of nutrition [2].

Omega-3 fatty acid (FA), a precursor for series 3 prostaglandins, is a long-chain polyunsaturated essential fatty acid and has been found to reduce platelet aggregation and inflammation and improve blood flow. Omega-3 FA suppresses the production of interleukin-6 (IL-6), tumor necrosis factor (TNF), and various leukotrienes, which are important mediators of inflammation [3]. Omega-3 FA supplementation is associated with accelerated patient recovery and improved survival in patients with peritonitis and abdominal sepsis [4].

Although studies prove the emerging role of Omega-3 FA in chronic abdominal infections, there is a paucity of literature on its effect on cases of perforation peritonitis. The present study examined the role of Omega 3 FA infusion in the surgical outcome of perforation peritonitis based on the hypothesis that it can modify the course of inflammatory diseases with a reduction in morbidity and mortality.

## Materials and methods

The study was conducted in a large tertiary care teaching hospital in India over a period of five years (January 2013-Dec 2018) and included 300 consecutive cases of perforation peritonitis undergoing surgery.

All the patients who presented with perforation peritonitis between the ages of 15 and 70 years were included in the study.

Patients who had hepatic dysfunction, renal dysfunction, diabetes, bleeding disorders, and known impaired lipid metabolism or documented hyperlipidemia were excluded from the study. Patients allergic to ingredients of parenteral 10% fish oil emulsion, and those who are on long-term immunosuppressive drugs were also excluded from the study.

The patients were divided into two groups of 150 each. The sample size in each group was calculated to keep the power of study at 80% with a 5% significance level. It was hypothesized that the wound complication rate will drop from 40% (the average wound complication rate in the authors’ institution in perforation peritonitis patients) to 10% after the use of Omega-3 FA supplementation. There was no selection bias, as patients were alternately assigned to each group. Data collection was carried out by an observer who was not aware to which group the patient belonged in order to eliminate observer bias. This clinical trial was registered in the Research Registry (research registry/7367).

**Figure 1:**
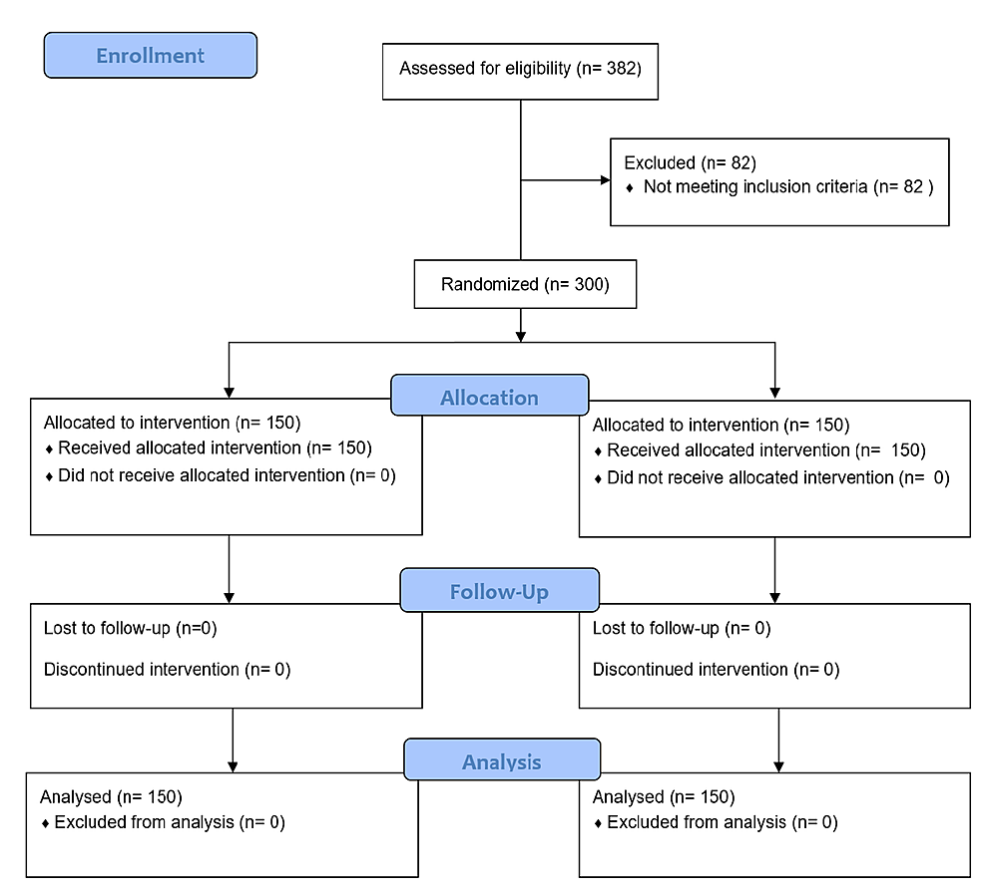
CONSORT flow diagram CONSORT: Consolidated Standards of Reporting Trials

The patients in the study group received an infusion of commercially available 10% Omega-3 FA emulsion (Omegaven, Fresenius Kabi Deutschland GmbH, India) in a dose of 2 ml/kg body weight for five days postoperatively. The preparation contains 10 g of highly refined fish oil in 100 ml of solution with 1.25-2.82g of eicosapentaenoic acid (EPA) and 1.44-3.09 g of docosahexaenoic acid (DHA). Patients in the control group received a placebo (normal saline) infusion in place of fish oil emulsion. No additional parenteral nutritional supplementation was given for the initial five days in any of the groups and patients in both groups were kept isocaloric through the infusion of dextrose-containing fluids. The Consolidated Standards of Reporting Trials (CONSORT) flow diagram is shown in Figure 1.

Peritonitis was defined on the basis of the clinical examination if the patient had generalized tenderness and/or rebound tenderness in the abdomen, guarding or board-like rigidity, and abdominal distension with absent bowel sounds. All patients who had hollow viscus perforation were included in the study. This was preoperatively determined by erect chest X-ray (CXR) or CT abdomen confirming pneumoperitoneum.

All the patients were started on antibiotics before starting the surgery in the form of intravenous amoxicillin-clavulanic acid combination (1.2 g, eighth hourly) with metronidazole (500 mg, eighth hourly) and continued for five days.

Written informed consent was obtained from all patients before inclusion in the study. Patients in both groups were matched in terms of age, sex, weight, and type of perforation peritonitis as shown in Table 1.

**Table 1:** Comparison of the baseline clinical parameters between the study and control groups TLC - total leucocyte count, CD - cluster of differentiation, CRP - C-reactive protein

Parameter		Study Group	Control Group	t-value	p-value
Age					
	15-30	21	31		0.16
	31-60	99	92		0.39
	>60	30	27		0.82
Sex					
	Males	109	105		0.62
	Females	41	45		0.56
Weight (Baseline) (Kg)		59.7±9.8	60.8±9.6	-0.98	0.33
Hematology (Baseline)					
Hemoglobin (g/dL)		12.2±1.8	12.7±2.7	-1.89	0.06
TLC (cells/mm^3^)		7967±3106	7688±3092	0.77	0.44
CD4 count (cells/mm^3^)		456±187	467±204	-0.48	0.63
Biochemistry (Baseline)					
Serum total proteins (g/dL)		5.51±0.99	5.54±1.07	-0.25	0.80
Serum albumin (g/dL)		2.94±0.76	3.01±0.78	-0.78	0.43
CRP level (mg/L)		45.6±22.3	47.1±22.0	-0.59	0.56
Serum cholesterol (mg/dL)		197±10.3	199±9.8	-1.72	0.08
Serum triglycerides (mg/dL)		147±14.2	144±13.6	-1.86	0.06
Cause of peritonitis					
Duodenal ulcer perforation		62	58		0.59
Tubercular perforation		36	40		0.69
Enteric perforation		21	18		0.61
Appendicular perforation		20	22		0.74
Traumatic perforation		7	6		0.80
Others		5	6		0.75

Preoperative evaluation

All patients underwent a preoperative work-up that included history and physical examination, including weight charting of the patient. In addition, the evaluation of hematological and biochemical parameters including haemoglobin (Hb), total leucocyte count (TLC), serum proteins, serum albumin, liver function tests (LFT), blood urea, serum creatinine, serum electrolytes, serum lipid profile, C-reactive proteins (CRP), and CD-4 count was also done.

Postoperative evaluation

Postoperatively, the following clinical parameters were observed in both the control and study groups, such as duration of hospital stay, resumption of oral feeding, duration of need for nasogastric tube aspiration, post-operative pyrexia, chest infection, wound infection, and weight.

All patients underwent laparotomy for hollow viscus perforation, as this was the standard protocol followed in the institute. A nasogastric tube was inserted routinely before the operation and the aspiration was continued postoperatively till the amount of aspirate was non-bilious and less than 100 ml/day or on return of bowel sounds. Postoperative pyrexia was defined as temperature ≥ 38ºC on two consecutive occasions, excluding the first 24 hours. Chest infection was defined as the development of pneumonitis or frank pneumonia based on clinical parameters like productive cough, pyrexia, and chest symptoms associated with reduced air entry, bronchial breathing, and/or crepitations on auscultation, which was confirmed with CXR findings. The postoperative wound was inspected daily by a blinded observer for any discharge (serous/purulent), erythema around the wound, gaping, or dehiscence (burst abdomen). Intraabdominal fluid was sent for cytology in all patients. Purulent discharge was defined as one containing pus cells on microscopy or growing bacteria in culture, otherwise, it was considered to be serous. Wound dehiscence was classified as partial if the rectus sheath was intact and complete if the rectus sheath was gaping along with evisceration of the gut. All patients underwent primary closure of the skin. The patients were weighed every third day to measure any weight loss/gain.

The laboratory parameters assessed were CD-4 count (evaluated on the third and sixth day), CRP (evaluated on the third and sixth day), serum total protein and albumin level (evaluated every alternate day), TLC (evaluated every alternate day), and serum lipid profile (on the sixth day).

The primary endpoint of the study was the status of wound healing on the seventh day. A healed wound ready for stitch removal was considered a favorable outcome, whereas the development of wound complications was considered an unfavorable outcome. The patients were discharged after healing of the wound and removal of stitches. The stitches were removed between eight and 10 days depending on the healing status. The secondary endpoint was non-abdominal postoperative complications and length of hospital stay.

Ethics

The study protocol was approved by the Institutional Ethics Committee of Maulana Azad Medical College ( F.No./11/IEC/MAMC/2012/246). All procedures performed in studies involving human participants were in accordance with the ethical standards of the institutional and/or national research committee and with the 1964 Helsinki declaration and its later amendments or comparable ethical standards. This research did not receive any specific grant from funding agencies in the public, commercial, or not-for-profit sectors.

Statistical analysis

Both the groups were compared for the various variables using the t-test (quantitative variables) and a chi-square test (qualitative variables). The difference was considered significant if the p-value was less than 0.05.

## Results

The patients in both groups were similar with respect to the demographic characteristics and the causes of peritonitis as summarised in Table 1. Overall, there were 214 male and 86 female patients (Male: Female = 2.49: 1). The age of the patients ranged from 15 years to 75 years.

Both the groups were compared with respect to several clinical as well as laboratory parameters.

It was seen that 82.67% of the patients in the control group developed postoperative pyrexia, whereas only 22.67% of the patients in the study group developed postoperative pyrexia (p = 0.0004). Only six % of the patients in the study group developed chest infections against 31.33% of patients in the control group (p = 0.0002).

**Figure 2:**
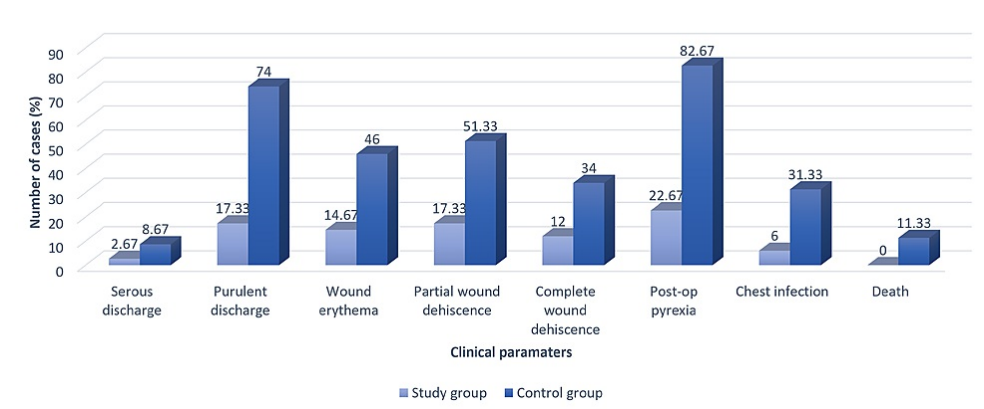
Comparison of the clinical parameters between the study and control groups

On the evaluation of wound healing, there was no significant difference in the presence of serous discharge from the wound in the two groups (2.67% versus 8.67%, p = 0.314), but there was a statistically significant difference in the presence of purulent discharge (17.33% versus 74%, p = 0.006), wound erythema (14.67% versus 46%, p = 0.004), partial wound dehiscence (17.33% versus 51.33%, p = 0.009), and complete wound dehiscence (12% versus 34%, p = 0.004) as shown in Figure 2.

**Figure 3:**
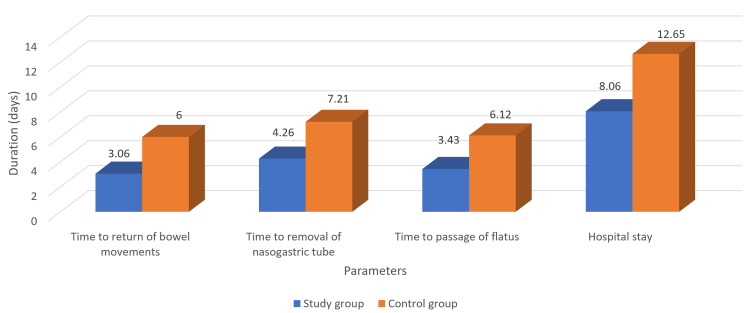
Comparison of the postoperative parameters between the study and control groups

The mean hospital stay in the study group was significantly shorter (8.06 days) compared to that in the control group (12.65 days) (p = 0.001). The mean time to return of bowel sounds (3.06 days versus 6 days, p = 0.002), removal of the nasogastric tube (4.26 days versus 7.21 days,p = 0.012), and resumption of bowel movements (3.43 days versus 6.12 days, p = 0.0021) were all significantly earlier in the study group compared with the control group as shown in Figure 3.

The weight of the patients showed an average fall of 1.08 kg in the control group compared with a rise of 0.15 kg in the study group (p = 0.032). Omega-3 FA is not directly related to weight gain in the study population. There was no mortality in the study group compared with 17 deaths in the control group (p = 0.0002). All the fatalities in the control group occurred within the first 15 days and were directly attributed to postoperative complications as shown in Table 2.

**Table 2:** Comparison of the post-operative parameters between the study and control groups * significant p-value

Complications	Study Group	Control Group	p-value
Non-fatal			
Serous wound discharge	4	13	0.314
Purulent wound discharge	26	111	0.0006^*^
Wound erythema	22	69	0.0004^*^
Partial wound dehiscence	26	77	0.0009^*^
Complete wound dehiscence	18	51	0.0004^*^
Intraabdominal collections	24	59	0.0008*
Pyrexia	34	124	0.0004^*^
Chest infection	9	47	0.0002^*^
Septicemia	5	2	0.218
Fatal			
Septicemia	0	15	0.0002^*^
Respiratory failure	0	2	0.157

On the evaluation of the hematological parameters in the two groups, it was seen that the mean hemoglobin concentration decreased by 5.7% in the first week in the control group while it increased by 2.76% in the study group (p = 0.001). The total leucocyte count increased by 6.2% in the control group while it decreased by 8.07% in the study group in the first week (p = 0.024). For the biochemical parameters, serum total proteins, as well as albumin, showed a fall in the control group (8.76% and 13.72% respectively), whereas these showed a rise in the study group in the first week (13.93% and 19.55%, respectively) (p = 0.002). The C-reactive protein levels showed a rise of 82.9% in the control group compared with a fall of 1.9% in the study group (p = 0.012). The CD4+ count showed a fall of 1.72% in the control group against a rise of 46.1% in the study group in the first week (p = 0.008). There were no significant changes noted in serum lipid profile in any of the patients.

## Discussion

Peritonitis is a commonly presenting surgical emergency that requires the use of both surgical and critical care skills for appropriate management. Perforation peritonitis is usually a result of peptic ulcer disease, acute appendicitis, enteric fever, tuberculosis, colonic diverticulitis, or trauma, which demands immediate surgical intervention, antibiotics, and critical care. There have been many postoperative complications of perforation peritonitis, especially in developing countries [1,3]. Current therapy in these cases leaves gaps for further improvement, considering the high mortality associated with the condition.

Omega-3 FA is known to exert anti-inflammatory properties, which are mediated by the uptake and assimilation of eicosapentaenoic acid (EPA) into the cellular substrate pool after oral ingestion. In states of inflammation, EPA is released to compete with arachidonic acid (AA) for metabolism by enzymes cyclo-oxygenase (COX) and 5-lipoxygenase. It also promotes the synthesis of lipid products called protectins and resolvins, which inhibit the production and regulate the migration of inflammatory cells and intermediates to sites of inflammation [4].

Omega-3 FA suppresses the production of IL-6, TNF, and various leukotrienes, which are important mediators of inflammation. The action of Omega-3 fatty acid begins instantly by exerting immune-modulating and organ-protective effects, even after short-term administration. Omega-3 FA administration can therefore modify the course of inflammatory diseases and significantly reduce morbidity and mortality toward the favorable side.

The metabolites of EPA have less inflammatory and chemotactic potency than substances derived from AA, which put a check on the full-blown inflammatory response. Omega-3 FA, specifically EPA, attenuates cytokine-mediated inflammation by various pathways like competitive inhibition and modification of mRNA of intermediary enzymes [4].

Omega-3 FA emulsion pretreatment is known to significantly attenuate lipopolysaccharide (LPS) stimulated macrophage TNF-α production in as early as four hours [5]. Omega-3 FA present in fish oil exerts its immune-modulating and organ-protective effects even after short-term administration. Its administration may significantly reduce mortality, antibiotic use, and length of hospital stay in different diseases [6].

Newer lipid-derived mediators called resolvins and protectins in animal models control the duration and magnitude of inflammation. They are derivatives of Omega-3 FA (eicosapentaenoic acid and docosahexaenoic acid) and possess potent anti-inflammatory, pro-resolving, and antifibrotic actions in vivo [7].

Omega-3 FA infusion reduces the severity of histopathology changes in acute pancreatitis and decreases lipid peroxidation in pancreatic tissue [8]. Omega-3 FA, on one hand, inhibits the formation of Omega-6 FA-derived pro-inflammatory eicosanoids (like prostaglandin E2 and leukotriene B4), and, on the other hand, can form several potent anti-inflammatory lipid mediators (e.g. resolvins and protectins). These, in combination with each other, suppress the activity of nuclear transcription factors, such as nuclear factor kappa-light-chain-enhancer of activated B cells (NF-κB), and decrease the production of pro-inflammatory cytokines like COX-2, TNFα, and Interleukin 1β [9]. Eicosapentaenoic and docosahexaenoic poly-unsaturated fatty acids (PUFA) may increase pro-inflammatory cytokine production at wound sites and thus provide a noninvasive, therapeutic potential to enhance cutaneous wound healing [10]. Immunostimulating parenteral nutrition, which incorporates Omega-3 FA, helps reduce the number of infectious complications, improves the function of the immune system, and has no influence on surgical complications, hepatic and renal function, and protein synthesis. Infusion of fish oil in doses up to 0.2 g/kg body weight/day has been proved safe regarding coagulation and platelet function, which are recognized adverse effects of Omega-3 FA [11].

In the present study, the demographic profile of patients in both groups was comparable in terms of the distribution of age, sex, and weight at the time of admission. The difference in the change in TLC of patients between the two groups after six days postoperatively was statistically significant (p = 0.024). Multiple studies support the anti-inflammatory role of Omega-3 FA. Lee et al. showed that EPA-derived lipid mediators possess markedly anti-inflammatory properties and the formation of platelet-aggregating factor (PAF) is reduced by EPA, which interferes with the precursor pool of PAF [12-13]. Fish oil improves the survival rate of animals after experimentally induced infections [14]. Omega-3 FA also improves the ability of monocytes to attach to bacteria [15-16].

The difference in the change of serum total protein and serum albumin of patients between the two groups six days postoperatively was also statistically significant (p = 0.002). The effect of 10% parenteral fish oil emulsion on these two parameters has not been studied in the previous studies. Since all the likely confounding factors in immunity and nutrition, i.e., age sex, weight, haemoglobin, serum proteins, and serum albumin at the time of admission were comparable between the two groups, it is plausible that the increase in the study group is as a result of the direct or indirect impact of 10% parenteral fish oil supplementation. This could, however, be also the result of variability in the measurement of albumin levels in stress versus non-stress states. Since serum albumin has also increased along with serum total proteins in the postoperative period in the study group, it is likely that this increase in serum total proteins was partly contributed by the increase in serum albumin.

Though the weight of patients in the study and control group were comparable on day zero, the difference in the weight change between the two groups was statistically significant (p = 0.032). There was a small increase in weight in the study group (0.146 kg) but there was a decrease in the control group of more than 1 kg. Since the intravenous fluids and medications given to the patients in both groups were comparable, it is likely that this increase in the weight of patients in the study group was not due to water retention. Moreover, the patients in both groups were kept isocaloric. A possible explanation for this is that the patients in the study group had fewer postoperative complications than patients in the control group and thus had a less negative nitrogen balance.

There was a decrease in the mean CRP level in the study group while it increased in the control group and the difference between the two groups was statistically significant (p = 0.012). Some studies have shown that Omega-3 fatty acid has a beneficial role in reducing the levels of CRP [17-18]. The difference in the change of mean CD-4 count in the study group compared with the control group in the present study was also highly significant (p = 0.008). Many studies, along with the present one, support the immune-enhancing action of Omega-3 FA [19-20].

The decrease in the length of postoperative stay in the study group was statistically significant when compared with the control group (p = 0.001). These results are comparable to the study of Weiss et al. [16]. In their study, the average postoperative stay in the intensive care unit was 4.1 days and 9.1 days in the test and control groups respectively, whereas the duration of the total clinical course was 17.8 days and 23.5 days in the test and control groups, respectively (p = 0.031). Also, many other studies have also shown that Omega-3 FA decreases hospital stay [6,21-24]. The decrease in the length of postoperative hospital stay in the study group may be a reflection of the improved nutritional status (increased Hb, serum total proteins, serum albumin, and weight), improved immune status (increased CD 4 count, decreased CRP), and less postoperative infection.

There was a significant early return of bowel activity in the study group compared with the control group. Consequently, there was an early removal of the nasogastric tube in this group compared with the control group. There has been no study to date to assess the effect of 10% parenteral fish oil emulsion on these parameters. Therefore, the role of Omega-3 FA in these parameters remains hard to explain.

Out of the 150 control group patients, 124 had postoperative pyrexia during some part of the postoperative period compared with only 34 in the study group during the same period. Pyrexia is a hallmark of infection in the majority of cases. This shows that Omega-3 FA protects from postoperative infections. There have been multiple studies in the past that have shown that pre/postoperative administration of Omega-3 FA decreases the incidence of infections [20,22,24-27].

While 47 patients (31.33%) had postoperative chest infections in the control group, only nine patients (6 %) had chest infections in the study group. Of the 47 patients with chest infections in the control group, 40 had severe infections in form of lobar pneumonia while the rest had pneumonitis. In contrast, only five patients of the nine patients in the study group had evidence of pneumonia. A report suggested that better control of the Omega-6/ Omega-3 PUFA balance may represent an interesting target in the prevention and/or control of *P.aeruginosa* infection in patients [28]. Another study reported that Omega-3 FA has a role in improving lung function in patients with COPD [29].

Multiple studies have supported the fact that parenteral/enteral fish oil consumption improves surgical/trauma-induced wound healing [3,30]. The inflammation induced by surgery is suppressed by the anti-inflammatory mechanism of the fish oil due to the decreased production of IL-1β, IL-6, and TNF by monocytes. Careful monitoring is required in people who are on antiplatelets as the Omega-3 FA reduces platelet activity.

In the present study, there was no observable change in the serum lipid profile (serum total cholesterol, high/low/very low-density cholesterol, and triglycerides) in any of the patients during the study period, thus obviating any adverse effects of the administration of FA emulsion on the lipid metabolism in the patients.

## Conclusions

The present study elucidates the beneficial effects of Omega-3 FA in patients with peritonitis. The Omega-3 FA helped in reducing postoperative complications and aids in an early, uneventful postoperative recovery. This was corroborated by both clinical as well as laboratory parameters. The authors, therefore, advocate Omega-3 FA as a promising tool for improving the postoperative outcome in critically ill patients. More studies are needed on similar lines to further clarify the role of Omega-3 FA supplementation in this subset of patients.
